# Value Propositions for Digital Shared Medication Plans to Boost Patient–Health Care Professional Partnerships: Co-Design Study

**DOI:** 10.2196/50828

**Published:** 2025-01-28

**Authors:** Benjamin Bugnon, Francesca Bosisio, Alain Kaufmann, Pascal Bonnabry, Antoine Geissbuhler, Christian von Plessen

**Affiliations:** 1 School of pharmaceutical sciences Institute of Pharmaceutical Sciences of Western Switzerland University of Geneva Geneva Switzerland; 2 CARA Association Épalinges Switzerland; 3 School of Engineering and Management Vaud HES-SO University of Applied Sciences and Arts Western Switzerland HEIG-VD Yverdon-les-Bains Switzerland; 4 The ColLaboratory - Participatory, Collaboratory and Action-Research Unit University of Lausanne Lausanne Switzerland; 5 Pharmacy Geneva University Hospitals Geneva Switzerland; 6 Department of Radiology and Medical Informatics Faculty of Medicine University of Geneva Geneva Switzerland; 7 Geneva Digital Health Hub University of Geneva Geneva Switzerland; 8 Department of Ambulatory Care Center for Primary Care and Public Health (Unisanté) University of Lausanne Lausanne Switzerland; 9 General Directorate for Health Canton of Vaud Lausanne Switzerland

**Keywords:** digital shared medication plan, medication records, medication list, e-medication, interoperability, electronic patient records, patient involvement, partnership, coproduction, medication safety

## Abstract

**Background:**

Health authorities worldwide have invested in digital technologies to establish robust information exchange systems for improving the safety and efficiency of medication management. Nevertheless, inaccurate medication lists and information gaps are common, particularly during care transitions, leading to avoidable harm, inefficiencies, and increased costs. Besides fragmented health care processes, the inconsistent incorporation of patient-driven changes contributes to these problems. Concurrently, patient-empowerment tools, such as mobile apps, are often not integrated into health care professional workflows. Leveraging coproduction by allowing patients to update their digital shared medication plans (SMPs) is a promising but underused and challenging approach.

**Objective:**

This study aimed to determine the value propositions of a digital tool enabling patients, family caregivers, and health care professionals to coproduce and co-manage medication plans within Switzerland’s national eHealth architecture.

**Methods:**

We used an experience-based co-design approach in the French-speaking region of Switzerland. The multidisciplinary research team included 5 patients as co-researchers. We recruited polypharmacy patients, family caregivers, and health care professionals with a broad range of experiences, diseases, and ages. The experience-based co-design had 4 phases: capturing, understanding, and improving experiences, followed by preparing recommendations and next steps. A qualitative, participatory methodology was used to iteratively explore collaborative medication management experiences and identify barriers and enabling mechanisms, including technology. We conducted a thematic analysis of participant interviews to develop value propositions for digital SMPs.

**Results:**

In total, 31 persons participated in 9 interviews, 5 focus groups, and 2 co-design workshops. We identified four value propositions for involving patients and family caregivers in digital SMP management: (1) comprehensive, accessible information about patients’ current medication plans and histories, enabling streamlined access and reconciliation on a single platform; (2) patient and health care professional empowerment through the explicit co-ownership of SMPs, fostering coresponsibility, accountability, and transparent collaboration; (3) a means of supporting collaborative interprofessional medication management, including tailored access to information and improved communication across stakeholders; and (4) an opportunity to improve the quality of care and catalyze digital health innovations. Participants discussed types of patient involvement in editing shared information and emphasized the importance of tailoring SMPs to individual abilities and preferences to foster health equity. Integrating co-management into the clinical routine and creating supportive conditions were deemed important.

**Conclusions:**

Coproduced SMPs can improve medication management by fostering trust and collaboration between patients and health care professionals. Successful implementation will require eHealth interoperability frameworks that embrace the complexity of medication management and support diverse use configurations. Our findings underscored the shared responsibility of all stakeholders, including policy makers and technology providers, for the effective and safe use of SMPs. The 4 value propositions offer strategic guidance, while highlighting the need for further research in different health care settings.

## Introduction

### Background

Lost or inaccurate medication information can cause patients and health care professionals significant difficulties [1-3] and lead to avoidable harm and costs [4-6]. Addressing these problems by improving timely access to and seamless communication of patient medication lists is a priority for medication safety everywhere [5,7]. However, personal, organizational, and contextual barriers often stand in the way, especially during transitions of care [8-10]. The growing burdens of chronic diseases and polypharmacy among aging populations add to these challenges. Thus, governments worldwide are investing in digital interoperability and data exchange systems to improve the quality of and access to information about patient medication lists [11].

Information systems in some countries support the management of digital shared medication plans (SMPs) based on treatment decisions and are usually embedded in patients’ electronic health records. These enable timely access to and updates of the list of medicines that a patient is currently taking by authorized health care providers. Some systems incorporate histories of recent changes in medication [12-14]. Other systems generate medication lists with administrative data from pharmacy dispensing records [15-17] or central prescribing databases [18]. The latter are less demanding for health care professionals but cannot ensure that the current treatment plan is up-to-date after changes have been made by patients, pharmacists, or other prescribers [18-20]. Furthermore, an SMP can encompass the administrative workflows of prescribing and dispensing [21]. The terms *plan* and *list* are used interchangeably in the literature. We prefer “plan” because it emphasizes the clinical focus on decisions and the active role of users. Patients and health care professionals can access plans through a web portal, a mobile app, or an established clinical information system. Health care professionals appreciate these systems [22-24], especially for medication reconciliation [25-27]. Digital SMPs have been implemented in Australia [28], Austria [23], Denmark [29], the United Kingdom [30], and Norway [26], among other countries.

Introducing a digital SMP poses significant challenges in health care settings worldwide, where fragmented and heterogeneous communication practices between health care professionals and patients are common. Switzerland exemplifies these challenges: prescriptions are the primary means of sharing medical orders but fail to account for changes when treatments are stopped. Moreover, medication plans are not consistently used by health care professionals and are often exchanged via email, fax, or on a piece of paper handed directly to the patient. This leaves patients largely responsible for managing their medication intake and sharing related information with health care professionals, relying on digital tools, handwritten or printed notes, or no tools at all.

Integrating a shared platform suitable for every actor is a complex challenge, which extends beyond ensuring medication data interoperability. Currently, despite the administrative, organizational, and management advantages of SMPs, medication list inaccuracies remain common because they are not systematically updated in health care services, over-the-counter medications are omitted, and patient-driven changes are inconsistently integrated [25,27,31]. Assigning the task of overseeing and updating medication lists can also be problematic. When general practitioners are solely responsible for this, specialist physicians, pharmacists, and nurses cannot document their changes and underlying reasoning because they can neither access nor edit the SMP [26,27,32]. Other systems require pharmacists to update SMPs when they provide medicines, give advice on over-the-counter medications, or conduct a medication review [23,33].

Currently, there are no national eHealth platforms that allow patients to change their medication plans independently [13,14,34], despite growing acknowledgment of how patients and families can contribute to improving medication safety [7,35,36]. Both digital and paper-based patient-held medication lists can strengthen patient self-management and enhance communication with their health care professionals [37-39].

This lack of patient involvement in established medication systems contrasts with the proliferation of smartphone apps for medication management [40] and web portals giving patients access to their clinical records and supporting their contributions to medication reconciliation [41-43]. This paradox should alert health technology developers and policy makers to the need for research and innovation in digital SMP design, use, and implementation. An SMP could leverage cooperation between patients and health care professionals to enhance the continuity of information and improve medication safety [14,27,44].

Some researchers have evoked the need to involve patients [25,27,31], but very few studies have sought out their opinions or tested the coproduction of medication plans [13]. Shifting to patient–health care professional coproduction would require considerable digital SMP redesigns in countries with established systems. However, Switzerland, having only recently introduced national shared electronic health records, known as “electronic patient records” (EPRs), has not yet implemented national e-medication or e-prescribing systems. One regional pilot project pointed out the poor engagement of patients whose SMPs provided no interactive features [14]. Finally, Switzerland’s eHealth interoperability framework provides an opportunity to design the digital capacity for coproducing medication plans and potentially inform similar developments in other countries [45].

### This Study

We aimed to explore and leverage the potential for patients’ contributions to SMPs. We used an experience-based co-design (EBCD) methodology to identify value propositions for a digital tool enabling patients, family caregivers, and health care professionals to coproduce and co-manage medication plans within Switzerland’s existing national eHealth architecture. We worked with polypharmacy patients, family caregivers, health care professionals, and digital health and quality experts.

## Methods

### Theoretical and Conceptual Framework

We used the coproduction in health care services framework model [46,47] and the Montreal Model [48] to embrace 3 types of coproduction: coproduction within our research team itself, coproduction to improve health care delivery, and coproduction during clinical interactions. Both models highlight the collaborative nature of health care services, emphasizing the need for greater patient involvement in research and innovation. The Montreal Model specifically underscores patients’ and family caregivers’ experiential knowledge. It describes their involvement as a continuum across various domains. Overall, the coproduction paradigm provides a valuable lens through which one can investigate the need for and benefits of collaboration between health care professionals, patients, and their relatives in daily practice.

### Research Team

The research team included a pharmacist with a master’s degree in health care service innovation (BB) and a physician with expertise in quality improvement, patient safety, and the coproduction of health care services (CvP). Both worked for the health authorities of the Canton of Vaud, one of the cantons making up the Swiss Confederation. Other members comprised a philosopher-ethicist, a health psychologist specializing in the sociology of technology (FB), and a sociologist (AK), all of whom worked at the University of Lausanne’s Participatory and Collaborative Action-Research Unit. There was also a physician specializing in digital health (AG) and a pharmacist specializing in medication safety (PB). The team had significant experience in qualitative research.

In total, 4 patients and 1 informal caregiver who had all participated in workshops about the rollout of a regional EPR system [49] were included as co-researchers in the study. They contributed to the study design; the preparation, facilitation, and debriefing of focus groups; and the writing and presentation of a synthesis for all the participants during the co-design workshops.

### Study Design

#### Overview

We applied the EBCD methodology in 4 phases [50-52] and conducted interviews and focus groups to develop “value propositions” for SMPs. Determining value propositions for new digital health tools is critical to their successful design and implementation. However, persistent misalignments between stakeholders’ views and the lack of measured evidence indicated that this task had often been overlooked in earlier projects [53,54]. Experts have argued that designing value propositions is a way of expressing how the development and implementation of a technology is worthwhile and a way of identifying for whom it creates value. Value describes what users or customers are attracted by (the demand side) and what benefits the solution can bring to their work, including its overall impact on the health system (the supply side). Value can have different meanings for different stakeholders and may involve trade-offs, such as the investment required to adopt and regularly use a tool. Furthermore, applying a service-design perspective to explore how different stakeholders understand a technology’s value proposition and its implications for their usual workflows can help rethink how health care services should evolve alongside the implementation of such digital solutions [54].

#### EBCD Phase 1: Capturing Experiences

In total, 5 patients and 1 family caregiver were interviewed individually to elicit their experiences of four common medication management situations previously identified through our literature review: (1) routine self-management using a medication plan, (2) patient-physician interactions about medications during consultations, (3) medication management after a major change in medication (eg, at hospital discharge), and (4) managing new drugs. Using their narratives and the literature, we developed fictitious but typical patient vignettes for each of the 4 key situations as the basis for initiating the ensuing focus groups.

#### EBCD Phase 2: Understanding Experiences

In total, 13 patients and 2 family caregivers were invited to participate in 2 parallel sets of focus groups (1 in Lausanne and 1 in Geneva). By discussing the 4 patient vignettes, the first focus group explored what “mattered” to these participants when they used a medication plan and collaborated with their health care professionals. We focused discussions on experiences and expected clinical outcomes and to identify key moments in the collaboration (touch points) that had significantly affected them. Participants’ questions and aspirations regarding a digital SMP were retained for the next phase.

A synthesis of the touch points identified served as the basis for initiating focus group discussions with 10 health care professionals. In a single, longer focus group, they discussed their understanding of patients’ and caregivers’ experiences and the potential for improvements by introducing a digital SMP (phase 3).

#### EBCD Phase 3: Improving Experiences

The same patients and family caregivers participated in 2 further parallel focus groups to explore potential improvements and problems that a shared digital tool might bring. The first part of each focus group provided participants with background information about Switzerland’s EPR systems and the policy context. In the second part, participants discussed how an SMP could facilitate the collaborative management of medication plans, with an eye to the 4 situations in phases 1 and 2. Participants were encouraged to describe the potential benefits of, enabling mechanisms for, and barriers to SMPs. Participants then gathered for the first co-design workshop to further discuss, reflect on, and synthesize their understandings and the potential for improvements due to the introduction of a digital SMP.

#### EBCD Phase 4: Preparing Recommendations and Follow-Up

Patients, caregivers, and health care professionals convened for the second workshop to discuss the synthesis of the results from the preceding phases and to make recommendations on developing an SMP.

Consistent with the principles of coproduction and the Montreal Model, we involved researchers and coresearchers in each step of the EBCD methodology, using iterative cycles of implementation, assessment, and adjustment to the approach and its associated documents. We aimed to create the best possible conditions for coproduction and patient involvement within both the project and future health care services using an SMP.

### Context and Setting

This study was conducted in the cantons of Vaud and Geneva in the Swiss Confederation’s French-speaking region between October 2020 and February 2021. Interviews, focus groups, and the EBCD workshops took place according to the COVID-19 regulations that were in place at the time and in calm settings at the University of Lausanne, Geneva University Hospitals’ innovation center, and Lausanne University Hospital.

The launch of a regional EPR platform for the secure storage and exchange of health data, as mandated by federal law, was in preparation in the region [55]. In total, 8 “communities” implement and manage EPRs in different regions of Switzerland. Currently, these EPRs function solely as repositories for clinical documents (Clinical Document Architecture level 1), generally PDFs, but the development of capabilities for sharing structured data within the national interoperability framework is underway. Medication and vaccination plans are priorities because of their implications for patient safety and clinical practice.

Our study was conducted in coordination with one of these communities, named CARA [56], which was piloting the development of a new SMP approach [57]. In cooperation with national bodies, it will apply international Integrating the Healthcare Enterprise pharmacy profiles [58] and the Swiss medication data exchange format based on the Fast Healthcare Interoperability Resources Foundation’s Health Level 7 specifications [59]. The architecture prepared by a formal national working group respects the patient-centered, decentralized design required by federal law. Technical details have been published previously [45].

The Swiss health care system is fragmented and has no national guidelines or policies for practices such as medication reconciliation and interprofessional communication. Legal reforms to safeguard the rights of polypharmacy patients to a medication plan and enhance medication safety have been proposed but have not yet been implemented, and the debate about them is ongoing [60].

### Participant Selection

Patients were invited to participate in the study if they (1) were capable of managing their medications autonomously (ie, they were not institutionalized), (2) regularly took ≥3 medications, and (3) had experienced transitions of care, such as hospital admissions and discharges that involved changes to medications. Family caregivers could participate if they regularly supported such a patient in taking medications.

Recruitment emails were sent to existing pools of volunteers affiliated with a regional consumer rights association, patients and family caregiver associations, and a local university hospital. The emails introduced the study topic and outlined the inclusion criteria. Once individuals had expressed interest to the concerned person in their respective organizations, the research team received their contact details and followed up via email or telephone, as preferred, to propose dates for the focus groups (scheduled 1 month in advance) and the co-design workshop with health care professionals (scheduled 2-3 months in advance). This follow-up step also confirmed their eligibility, interest, and availability.

We aimed for diversity of experiences, diseases, gender and age. To achieve this, we also contacted individuals already involved in existing initiatives directly, such as peer support, teaching, or research projects. Our initial goal was to organize 3 to 5 local groups of 5 to 9 participants each, for a total sample size of approximately 15 to 30 individuals.

The inclusion criteria for health care professionals were (1) previous participation in improvement projects on medication management, transitions of care, or care coordination; or (2) involvement in medication prescription, delivery, or management in their current occupation. They were recruited through the professional networks of the authors.

### Data Collection

Data were collected through individual interviews, focus groups, and workshops with patients, caregivers, and health care professionals per the 4 phases of EBCD. Guides were prepared for each phase by the research team and refined between interviews (Multimedia Appendix 1). Focus groups in phase 2 were based on the patient vignettes built up from the available literature and narratives collected in phase 1. The focus groups with health care professionals were guided by the key touch points revealed by the focus groups with patients’ informal caregivers.

At least 1 coresearcher participated in each focus group, asking follow-up questions and taking notes that were shared with the team. Coresearchers participated in preparing and debriefing each focus group and workshop during team meetings. The division of tasks is provided in the Authors’ Contributions section.

### Data Analysis

 We conducted an in-depth thematic analysis of our transcriptions per the recommendations of Braun and Clarke [61]. Two researchers independently coded the different series of patient focus groups in parallel. They compared codes and discussed disagreements regarding the raw data until they reached a consensus. One then finalized the coding for the 5 focus groups. Subsequently, we developed themes (also using personal notes and intermediate outputs from the co-design process) that had repeatedly been raised, discussed, and validated by the research team and by the workshop participants. The review, definition, and final naming of the themes were done iteratively by the authors. Analyses were structured using MaxQDA software (VERBI GmbH). We followed the COREQ (Consolidated Criteria for Reporting Qualitative Research) guidelines [62].

A professional interpreter translated selected citations for this paper from French to English. Bilingual team members verified the content.

### Ethical Considerations

Our regional ethics review board formally confirmed that it did not need to review and approve the study, as per the Swiss Federal Human Research Act (Req-2020-00591). Each participant received oral and written information about the study and signed the consent form before participation. The consent form specified that, after recording, transcripts would be deidentified, and no personal statements would show names for any purpose. To ensure a safe and open environment for discussion, participants were asked not to share specific sensitive personal information; instead, they were encouraged to draw on their experiences to guide their contributions. At the beginning and end of each discussion, participants were reminded to ensure the confidentiality of the content shared. All data were securely stored within the research university’s information system. Transportation costs were reimbursed according to university guidelines based on public transport fares. Parking costs at the university site were also covered. No other financial compensation was provided; however, participants were offered an aperitif after the workshop.

## Results

### Participants and Data

Between August and October 2020, we recruited 31 individuals (patients: n=18, 58%; caregivers: n=3, 10%; health care professionals: n=10, 32%) with a broad range of experiences regarding medication management plans from a variety of care settings (Table 1).

We formed 2 local groups of patients and caregivers, one less than initially planned, but COVID-19 complicated the recruitment of people with respiratory diseases.

Individual interviews in phase 1 lasted from 43 to 71 minutes. Focus groups in phases 2 and 3 lasted from 115 to 130 minutes, and EBCD workshops lasted from 120 to 210 minutes. Table 2 summarizes the participation in each phase of the EBCD workshops. Three individual interviews were conducted as a backup for participants who could not attend a focus group.

**Table 1:** Focus group and interview participant characteristics.

Characteristics		Patients^a^ and caregivers (n=21)		Health care professionals (n=10)^b^
**Gender, n (%)**				
	Women	7 (33)	6 (60)	
	Men	14 (67)	4 (40)	
**Age range (y), n (%)**				
	36-50	4 (19)	8 (80)	
	51-65	10 (48)	1 (10)	
	66-78	7 (33)	1 (10)	

^a^Health conditions were autoimmune, blood, musculoskeletal, gastrointestinal, rare neurological and mental health diseases, as well as cancer, and diabetes. One person had undergone a renal transplantation.

^b^The clinical backgrounds of the 10 health care professionals were medical secretary working as case manager 1 (10%); 2 (20%) nurses in gerontology and primary care; 3 (30%) community and hospital pharmacists; and 4 (40%) physicians in hospital internal medicine and general practice.

**Table 2:** Participation in focus groups and interviews related to the phases of experience-based co-design (EBCD).

EBCD phase	Type of interview	Participants
Capturing experiences (phase 1)	Individual interview	6 patients and caregivers
Understanding experiences (phase 2)	Focus group	15 patients and caregivers divided into 2 groups and 1 group of 10 health care professionals
Improving experiences (phase 3)	Focus group with individual interviews as backup	Same groups as phase 2
Improving experiences (phase 3)	First EBCD workshop	All 31 participants together
Recommendations on improving experiences and follow-up (phase 4)	Second EBCD workshop	All participants were invited: 19 patients and caregivers and 10 health care professionals

The subsequent sections highlight the main results from our analysis of the discussions with participants in phases 1 to 3, summarized in Textbox 1. Recommendations for action codeveloped with participants during phase 4 are briefly described in the Recommendations for Action section, alongside the value propositions.

**Summary of the value propositions for digital shared medication plans (SMPs).**

**Comprehensive and accessible information about patients’ current medication plans and histories**

- Streamlined access and transmission of medication information
- Shared comprehensive medication information going beyond prescriptions
- Reconciled medication information using a common platform

**Patient and health care professional empowerment through the explicit co-ownership of medication plans**

- Shared responsibility for medication management plans is made explicit
- Defined depth of patient involvement in editing the information shared
- Enhanced visibility of the contributions to building an accountable interprofessional team

**A means of supporting collaborative medication management**

- Enhanced joint planning, execution, and monitoring using a medication plan
- Tailored access to medication information within the SMP
- Facilitated interprofessional coordination with lower patient and family burdens

**Quality improvement and innovation**

- Strengthened care partnerships
- Improved integration of care, efficiency, and patient safety
- Catalyzation of digital health innovations

### Value Propositions for the Joint Management of Digital SMPs by Patients and Health Care Professionals

The thematic analysis of each value proposition for the joint management of SMPs resulted in 4 themes and their subthemes, as summarized in Textbox 1.

#### Comprehensive and Accessible Information About Patients’ Current Medication Plans and Histories

Participants emphasized the importance of having digital medication plans and histories on a common eHealth platform, where information is accessible, complete, and regularly updated. The added value lies in the information mentioned subsequently.

##### Streamlined Access and Transmission of Medication Information

The continuity of information transmission is key throughout patients’ care trajectories. That transmission often depends on a patient or a caregiver acting as the link (patient, focus group, Lausanne 1). This was perceived as being a major burden on them. In addition, information transfer is at risk when patients cannot fulfill this task:

So, for me, I’ve...I see a rheumatology specialist for my polymyalgia, and I realize that afterwards, when I consult my doctor, my GP, well, it’s me who has to tell her everything I’m taking, everything the other doctor did, et cetera. So, it works very well, because I make the link. But I don’t understand why we still don’t have that electronic patient record and other stuff containing all the information, so that the doctors you give access to—because you have to give them access—can see what’s going on for themselves and intervene if necessary. It seems like an essential project, to me.

Patient, focus group, Geneva 1

Health care professional communication with patients is mainly oral, except for written prescriptions and, in some cases, a medication chart. This was problematic for some patients, especially if they were taking many different medications over long periods and these were frequently modified:

[With regards to healthcare professionals not communicating with each other], the patient is there in the middle and just has to get on with it...must sort out their emotions and then make some sense out of all those words, and the jargon, and the protocols, and the processes that they’ve been given, and then, what’s more, they’ve got to try to understand...

Patient, focus group, Lausanne 1

Patients develop and use tools that help them in their roles as transmitters of information, such as taking photographs on their smartphones “to remember names” (patient, focus group, Lausanne 1), making lists on their computers (patient, interviews 3 and 4), or keeping printouts in their wallets (patient, interviews 2 and 5). However, these tools are unreliable in emergency situations or during travel, when access to them is not guaranteed and their validity cannot be checked. Secure web-based access to precise information about a patient’s current medications and a history of their modification could provide a practical tool that embraces patients’ key role in transmitting information, with potentially major improvements to patient safety.

##### Shared Comprehensive Medication Information Going Beyond Prescriptions

Prescriptions are usually available in writing, yet they only include a fraction of the information required for medication management:

A prescription might only be partial; a final treatment plan should really summarize all the medications that patients are taking: the medications that are prescribed, but sometimes also those that aren’t prescribed and that have been ordered online, as you said, or lastly, self-medication, and alternative and complementary medicines.

Nurse, focus group, health care professionals

Major deficiencies in information include missing not only indications or justifications for prescriptions, dose adjustments, and cessations of medications but also diagnoses, laboratory values, or drug allergies, none of which is usually included in prescriptions, in communications with patients, or between all the health care professionals involved.

##### Reconciled Medication Information Using a Common Platform

An SMP enables the reconciliation of all the information from all the contributors to a patient’s medication in a single location. Health care professionals can thus rapidly find useful information that is particularly relevant during transitions of care and emergencies:

The patient leaves hospital with their prescription, arrives at the community pharmacy, and then there are a certain number of interactions that take place there, questions, and they can’t answer them or fill in the missing information...The assistant physician isn’t contactable, so they’ll call the treating physician. But it’s Saturday...So, because of this fragmentation, it becomes indispensable for everybody to be available.

Pharmacist, focus group, professionals

Health care professionals highlighted that the necessity to regularly update an SMP depended on its use being appropriate to the setting and context, including aspects of the information systems used (eg, interoperability), the clinical processes in place (eg, trained staff), and the framework conditions (eg, financing and legal duties).. Health care professionals hoped for an SMP that would simplify their daily practice and be user-friendly. Digital technologies also introduce additional concerns about data security and confidentiality.

#### Patient and Health Care Professional Empowerment Through the Explicit Co-Ownership of Medication Plans

Participants recognized the intrinsic coproduction existing between patients, caregivers, and health care professionals preparing and using medication plans. They emphasized the importance of empowering individuals to fulfill their roles in this coproductive effort and boosting their sense of shared ownership.

##### Shared Responsibility for Medication Management Plans Is Made Explicit

The patient, family caregivers, and health care professionals already “share responsibilities” (patient, focus group, Lausanne 1) for the continuity of information transmission and for being “on the same page” (patient, interview 2), with or without an SMP. Patients must share their health information with health care professionals, who, in turn, must obtain medication information, document interventions, and communicate with their patients. Pharmacists verify prescribed medications and explain appropriate medication use during dispensing to ensure safe medication practices. Patients are ultimately responsible for taking their medication, whereas family members may assist or “negotiate” administration and intake (family caregiver, interview 5). Both health care professionals and patients make decisions and act on information, but patients are the most affected by the outcomes.

An SMP can increase transparency and contribute to raising awareness of the importance of communication about medications between patients and their health care professionals. However, it requires open, trusting, and caring relationships for patients not to modify or discontinue their medication without informing health care professionals:

In an electronic patient record, if they don’t take [their medication], you should be able to see that fairly easily, theoretically. They won’t be judged, but you’ll be able to tell whether they are able to follow the guidelines. They have every right to stop [their medication].... They should be able to discuss this easily with the professional...

Physician, focus group, professionals

Furthermore, an SMP giving the relevant stakeholders the right to view and update shared information could empower patients and health care professionals to develop a shared sense of responsibility for medication management. The traceability of the authorship of modifications is crucial in this regard. Assuming joint responsibility could improve how different stakeholders learn from each other, leveraging their respective resources and building mutual trust in their collaborative partnership. The opportunity to participate could balance patient-health care professional power dynamics and increase patient autonomy:

...once that responsibility has been rebalanced and truly shared, I think that, well, trust should come as a matter of course. Because if the patient has come far enough, is sufficiently mature to realize that it’s for their benefit, if the physician has sufficient trust that their patient is a stakeholder in their treatment management, in their healthcare trajectory, well, then there’s no need to discuss sharing responsibility because everybody’s got some...

Patient 1, focus group, Lausanne 1

The patient has also got to have their share of responsibility, because when you feel responsible, you feel like getting involved.

Patient 3, focus group, Lausanne 1

Thus, the co-ownership of an SMP provides practical ways of partnering and assuming shared responsibility for medication management plans.

##### Defined Depth of Patient Involvement in Editing the Information Shared

Discussions on the breadth of possibilities for patients and family caregivers to update an SMP were recurring. Given that patients are the end users of medications, it seemed relevant that they could document changes and rapidly report self-medication in an SMP themselves. Such access would also enable patients to verify their current medication plans and rectify any communication errors made by health care professionals, potentially preventing harm. Similarly, health care professionals could identify and correct errors, ensuring that medication plans are up-to-date and accurate. In contrast, patients having editing access also raised concerns about introducing new errors or causing adherence problems. The debate for and against patients’ editing rights is well described in this discussion:

If there’s no legal basis for it, well, it can’t work...it [will be]...the law of the jungle, because if everybody goes off on their own, adding everything and anything, that can be dangerous too if the poor physician at the emergency department finds that everything’s been modified.... If they want to stop a medication, well, me, I’d telephone my physician. But I wouldn’t document, “Well, I’m stopping,” off my own bat. Like you said, we’re not doctors.

Patient 1, focus group, Geneva 2

I see it exactly in the same way.

Patient 7, focus group, Geneva 2

For people who’ve been taking the same treatment for a long time, I think things are different because you know very well how you react. Your physician knows very well that sometimes you get fed up.... I think that it’s good that you’re able to do it and to inform the practitioner.

Patient 6, focus group, Geneva 2

Participants agreed that clear responsibility for changes and their consequences was needed. Ideally, each partner should contribute to and share in that responsibility. At the same time, joint management of an SMP places a significant responsibility on patients, and their level of involvement must align with their personal resources and preferences. Thus, joint management should be a right and an ideal to strive for rather than an obligation. Likewise, health care professionals should be well-trained and well-equipped. “Ethical and legal questions” (pharmacist, focus group, professionals) include careful consideration of health care professionals’ responsibilities, the confidentiality of sensitive information, and situations where patients choose to or are incapable of transmitting information and sharing responsibility for medication management planning. These questions are intimately linked to health policies and legal requirements:

But in some precise cases, can we make it obligatory? That’s to say, me, for example, when it comes down to it, I’m aware of it, so, in the end, I’m for this record. I’ll even push all my physicians to complete it because I think it’s pretty important. But couldn’t somebody who’s losing their marbles a little bit...in this particular case, couldn’t it be made obligatory for them, and for their physicians to do all this follow-up?

Patient, focus group, Geneva 2

As a compromise, participants proposed that patients’ and family caregivers’ editing rights could be activated flexibly or be confined to the medication they have added, such as self-medication. Furthermore, they emphasized that an SMP solution should support health care professionals and patients in fulfilling their responsibilities through, for example, cues and reminders about medication reconciliation.

##### Enhanced Visibility of the Contributions Toward Building an Accountable Interprofessional Team

SMPs have the potential to stimulate interprofessional and patient collaboration by enabling better visibility of the contributors and their actions, thereby fostering a sense of accountability. SMPs promote transparency and encourage active participation, making everyone’s contributions visible and tangible. However, it is important to acknowledge that this transparency may encounter some resistance among health care professionals due to concerns about their legal exposure and the potential disregard of their clinical judgment by patients or peers. Similarly, patients might not trust health care professionals or the health care system itself, and they may not want every detail of their EPR to be available to every health care actor. Nevertheless, participants agreed that information sharing was crucial to effective interprofessional collaboration and patient-centered care:

Well, the electronic patient record and this medication management and whatnot, et cetera, got me interested straight away, and I said to myself, “Well, there’s really something to be done here.” Finding solutions isn’t straightforward because you have to get healthcare specialists to talk with each other and to speak a common language. Because, very often, they’ve each got their own jargon, and the specialist will say, “Anyway, I did not study gastroenterology, so it’s not directly my problem.” Or often, in my case, I hear, “It’s due to the diabetes.”

Patient, focus group, Lausanne 1

Patients stated that having everyone working for and with them, as a “team,” was a great privilege. Team members using an SMP might have more clearly apparent bonds thanks to shared, transparent information (patient, focus group, Geneva 1 and 2).

#### A Means of Supporting Collaborative Medication Management

According to the study participants, an SMP is a means to develop and support collaboration in daily practice.

##### Enhanced Joint Planning, Execution, and Monitoring Using a Medication Plan

Participants perceived SMPs as valuable aids in preparing for consultations with health care professionals and for use with them during these interactions. These tools should be designed and implemented to enhance reviews of and communication about medication:

Well, it’s a reminder. I mean to say, when I get to the doctor’s, it’s kind of my roadmap. We’ll open it up together. We’ll say, “Well, so, how’s it going? Have these medications here been taken? Oh, look, so you’ve got a new medication?” Or, in my case, “Oh, so you’ve stopped this medication?” Well, to start with, you get yourself into the situation. I think it’s a good place to start...

Patient 4, focus group, Geneva 2

What’s important is that you said, “Open it up together,” you see?

Patient 2, focus group, Geneva 2

SMPs could also increase medication follow-up by supporting patient self-monitoring and management as well as interprofessional communication. This could be particularly important when dealing with major changes, such as a hospital discharge:

It’s certain that the time for preparing a [hospital] discharge goes by pretty quickly, and we have to manage the patient’s medications right up to the end [of their stay], ... we completely take over their role. If this tool [an SMP] could be used several days before the discharge...with the treatment management plan updating itself, we could also end up evaluating the patient’s true level of understanding a few days before their discharge, and whether they’ll be able to get by with their medications.... And then we could implement the proper interventions.... That really could be super interesting at care transition time.

Nurse, focus group, professionals

Participants suggested that SMPs could also help existing coproduction practices, such as negotiating a “break” from usual medications (patient, focus group, Geneva 2) by checking boxes next to vital medications. SMPs could include action plans for rescue medications, such as for “...antibiotics. I know exactly when to take them and at what dosage. I inform (my treating physician) afterwards” (patient, focus group, Lausanne 1). Finally, SMPs could foster discussions about medicines and encourage regular reviews of medication management plans by clinicians, as this patient described the following:

Every two consultations, I ask the physician, “Which medications could we eliminate?”[Patient, focus group, Lausanne 1]

##### Tailored Access to Medication Information Within the SMP

The same medication information, held within an SMP, could be presented in a manner tailored to each user, health care professional, or patient. Personalization according to patient preferences and different users’ levels of health literacy would thus be possible. These functions would help patients to more easily remember the medications they want to discuss with their health care professionals:

...when I go to a new physician and he asks me which medication I take, well, I take photos of my medication boxes, because one time in ten I’m incapable of either pronouncing the name or remembering what I’ve got to take. For me, it’s just the green pill.

Patient, focus group, Lausanne 1

Furthermore, an SMP platform could improve medication safety by giving advice, preventive messages, and explanations. Health care professionals could also use SMPs to personalize the written information patients receive about their medication use and, importantly, to ensure that interprofessional communication is more consistent. The platform could also help to provide treatment options and possibilities for shared decision-making. Although everyone should have access to information about their medications, the technical level of the information provided needs to be tailored to individuals’ needs, capacities, and expectations. The inclusion of pictograms, videos, and translations into different languages might help to meet patients’ diverse needs. Tailored and flexible features, rights, and decision-making aids could help to create equitable medication management systems.

##### Facilitated Interprofessional Coordination With Lower Patient and Family Burdens

Communication gaps and fragmented documentation hinder coordinated, collaborative care. Using SMPs could improve this by including the reasons why a medication needs to be taken and ensuring that instructions about medications align with the recommendations of different health care professionals, as a pharmacist highlighted the following:

...typically, the patient should have properly understood that, despite the side-effects or the drug-drug interactions, the physician wants to try it [the newly prescribed treatment] out for two weeks, and that they [the patient] have thus accepted [the risk]...even though they’ll have to answer [the question about the treatment decision] again [at the pharmacy], because we’ll ask them the same question, just using other words...probably...which can cause some confusion, unsettle the patient, and increase the risk of giving contradictory information.

Pharmacist, focus group, professionals

Furthermore, patients and health care professionals expect SMPs to facilitate planning and discussions between different health care professionals, allowing for more consistency and coordination in the treatment:

So, the advantage of a medication plan—because a medication plan means that you’re also planning a treatment—and because that plan is available to all the specialists, because it’s electronic, well, so, its advantage is that the specialist can, at any given moment, ask questions, because not every specialist necessarily knows what medications the patient is taking.

Patient, focus group, Lausanne 1

Finally, SMPs could decrease the coordination burden for patients and family caregivers, thus reducing the risks of disengagement or distress:

Because you’re fighting and struggling with each of the physicians, at the pharmacy, at the hospital...repeating the same info, explaining why the plan isn’t a standard one but is the best suited to you...What’s more, you have to convince [them] that you know what you’re talking about, because, yes, there are some drug-drug interactions, but it’s the combination that has suited me best for a long time...After a while, you just feel like letting everything go to hell—giving up on everything.... Me, I’m not at all surprised when you read in the papers that 50% of the medications prescribed don’t get taken and when you hear that therapeutic adherence is a real problem.

Patient, interview 4

#### Quality Improvement and Innovation

SMPs provide new opportunities and can enable quality improvement and innovation.

##### Strengthened Care Partnerships

Participants highlighted the growing interest in “health partnerships” (patients, focus groups Lausanne 1 and Geneva 1), emphasizing that SMPs not only enable patients and health care professionals to partner around a medication plan but also promote a more collaborative health care paradigm:

...you should explain it to them from the outset, because afterwards, when you’re using the tool, you’re obviously going to have to work in partnership with them.

Patient 7, focus group, Geneva 2

It’s all about a change in mentality.

Patient 2, focus group, Geneva 2

##### Improved Integration of Care, Efficiency, and Patient Safety

SMPs can improve efficiency, patient safety, and the integration of care. Nevertheless, the added value of an SMP depends on a favorable context and well-executed implementation. Participants emphasized the importance of promoting and then managing change. Incentives, including legal obligations, were mentioned several times:

So, obviously, among the barriers, there’s time. The time it takes to fill in all the information. Who’s the guarantor of that information? What competencies do you need? And who reimburses us for doing it?

Pharmacist, focus group, professionals

It’s like any change in your life. Change is hard; it takes a certain amount of time to adapt.

Patient, focus group, Geneva 2

Health care professionals emphasized that SMPs would be particularly beneficial when combined with clinical interventions such as medication reconciliations, medication reviews, care coordination by a case manager, patient education, or support for medication self-management.

##### Catalyzation of Digital Health Innovations

SMPs could serve as springboards for creating and scaling up digital solutions for patients and data-driven innovation. Augmenting the platform with additional features could help patients in their medication self-management and foster better communication with health care professionals, for example, by tracking medication intake and symptoms. Furthermore, leveraging data from an SMP could stimulate innovation and bolster research, pharmacovigilance, and other continuous improvements:

I’d add...and clinical research. Because medications are tested one compound at a time, if you like, then in an age when you’ve got multimorbid patients who’ve got several types of medications to take, there’s no clinical research on the cumulative side-effects of these different medications, and shared medication plans could be an extremely rich source of information.

Physician, focus group, professionals

### Recommendations for Action

During the final co-design workshop, participants reached a consensus on three key actions to advance toward the joint management of SMPs: (1) the cocreation of an accessible and empowering platform for SMPs that accommodates diverse patient population groups, (2) the promotion of best (clinical) practices that emphasize the use of collaborative SMPs with patients and health care professionals working in partnership, and (3) stakeholder dialogues to establish the necessary enabling environment.

## Discussion

### Principal Findings

**Figure 1:**
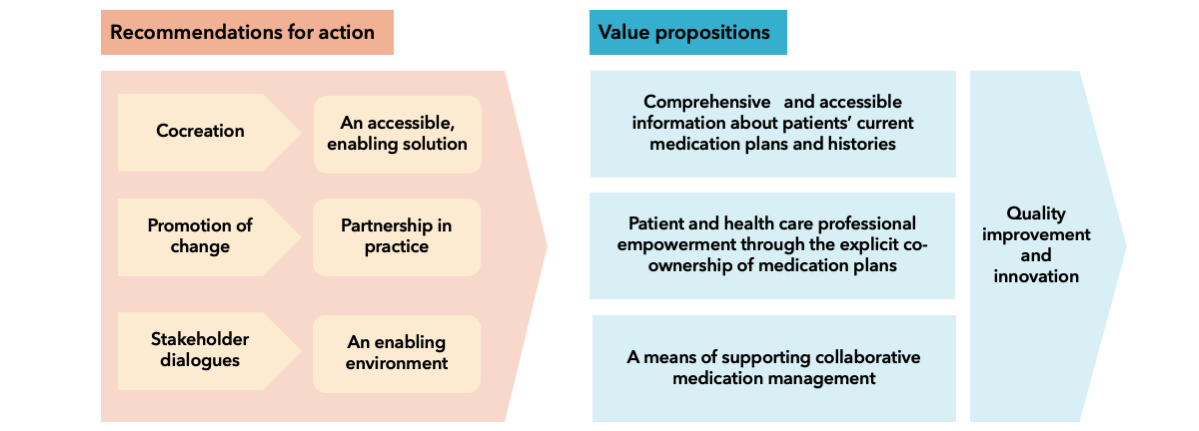
Summary of the value propositions for digital shared medication plans and the actions recommended for their implementation.

Our findings underscored the importance of explicitly recognizing and promoting the co-ownership of medication plans. The value of digital SMPs lies in making it easy for patients, family caregivers, and health care professionals to create and update medication plans, for example, via the possibility of adding over-the-counter medications. Apart from improving the quality and safety of medication management, this could strengthen interprofessional and patient collaboration, enhance medication self-management, and facilitate innovations in care coordination and medication safety. To succeed, the co-management of medication plans must be integrated into clinical practice and supported by interactive information systems that can be tailored to individual capabilities and preferences. The value propositions from our analysis and the recommendations for action defined by the participants are summarized in Figure 1.

The core value of digital SMPs lies in facilitating the navigation of a patient’s current medications and medication history. Both patients and health care professionals would benefit from a clear overview of recent changes and the possibility of distinguishing between changes made by the patient and health care professionals. Additional features, such as reminders to administer medication, self-management guidelines, patient education resources, self-monitoring tools, and secure messaging, could further enhance the practical and safety values of such systems. For patients who might be less comfortable updating their medication plans alone, guided assistance should be provided, such as scheduling medication reviews or reconciliation appointments where a health care professional can verify and upload information. Preparing a well-structured, shared outline of how these appointments might work could enhance patient involvement and empowerment, improving the efficiency of clinical interventions. Certain digital patient mobile apps offer some of these features [40,63] and could be incorporated into a web-based SMP platform for patients that would facilitate effective collaboration between them and health care professionals.

### Value Propositions

Our findings challenge the prevailing prescriber-centric paradigm of existing SMP platforms that do not ensure the accuracy and safety of medication information. For example in Denmark, a world leader of digital medication information, 78% of hospitalized patients had at least 1 discrepancy between their actual medication intake and the documented list in the national shared record that can be accessed by health care providers. Nearly half of these discrepancies were due to changes made by patients, that were not known and registered by the physicians [31]. More recent initiatives in neighboring Nordic countries continue to use SMPs that limit active contributions of patients [21]. Once we understand the limitations of SMPs managed solely by physicians [24,27], a more collaborative approach seems to be worthy of further exploration.

The co-management of SMPs could be a game changer in ensuring the accurate transfer of information at care transitions, enabling synergies, and benefitting from the accumulated efforts of all the stakeholders. Reconciling discrepancies in medication lists and dealing with their consequences cost health care professionals precious time [1,8]. An SMP would facilitate information flows along patients’ clinical trajectories [18,26,64]. Information system interoperability, supportive digital functionalities, and patient involvement are known facilitators of broad-based medication reconciliation [8,65,66]. Accordingly, the World Health Organization promotes collaborative medication management involving patients and their families as partners [7]. Nevertheless, determining whether SMPs effectively reduce discrepancies requires further research and evaluation.

Patient-held medication lists are widely endorsed as a strategy to improve medication safety [7,37]. Patients actively manage and communicate medication information, and they prevent and mitigate medication errors [2,35,67]. Compared with other patient tools [37,63], the added value of an SMP lies in its 2-way link between patients and health care professionals and in the secure web-based storage of current medication lists and histories of changes. A partnership with patients that goes beyond holding lists could enhance the effects of such systems [36,68].

Indeed, an expanding body of evidence supports the argument for patients managing their medication plans. Patient-held medication lists have made them feel empowered and increased their self-confidence [22,37,39]. Involving patients in digital medication processes has facilitated medication reconciliation [63], saved time, and reduced medication errors [66,69,70]. Likewise, access to clinical notes has benefitted communication, trust, and medication adherence [71-73]. One quasi-experimental study showed that giving patients access to shared records through a platform integrating their interactions with health care professionals improved medication adherence [71]. The ability to edit lists seemed to be more motivational than read-only access [14,34].

Notwithstanding the potential advantages of shared medication lists [38], their implementation requires very careful attention. Variable levels of health literacy and a general lack of engagement are recognized as barriers to implementation and use. In one German study [74], <50% of patients had a comprehensive understanding of the medication plan that their general practitioner was legally obliged to share with them. Thus, strategies for medication management must be thoughtfully designed and implemented to accommodate diverse users and preferences [63]. Co-designing systems with the aid of patients with diverse backgrounds and integrating artificial intelligence solutions could prove pivotal to the successful adoption of such tools and may help avoid any unintended exacerbations of health inequalities due to digitalization.

We argue for a system design that empowers the collaboration of all the stakeholders in medication management. Such an approach needs effective leadership and change management to accompany the required organizational and sociocultural adaptations to clinical practice. In processes like this, trust between stakeholders and in the technology is critical for successful system implementation and use [14,75]. However, trust cannot be decreed. Notably, the inability to correct obvious errors in a medication list may create mistrust [76]. Finally, a shared platform may promote good practices and aid advocacy for medication safety being “everyone’s business” [77]. SMP systems involving every stakeholder can be disruptive, and we hope that our value propositions will encourage experimentation and open innovation in the field.

### Strengths

By engaging with patients, caregivers, and health care professionals, we leveraged coproduction and diverse participant experiences to elicit innovative value propositions for a digital SMP system. Collaborating with coresearchers and a multidisciplinary research team provided complementary perspectives and enhanced reflexivity throughout the study. Exchanges within parallel groups, composed of participants with profound experiential and professional knowledge, enriched the discussions on medication management. Experienced participants were rapidly able to contribute effectively to the focus groups and EBCD workshops, motivated by the rare opportunity to discuss with both patients and health care professionals. In future codesign initiatives, we recommend including additional meetings with participants if fostering group dynamics and collaborative engagement requires more time. Interestingly, our approach cultivated a sense of shared responsibility among the participants, as observed in earlier co-design processes [78]. Most (21/31, 68%) of the participants have since continued working on the implementation of SMPs and EPRs in different advisory and networking groups.

### Limitations

One limitation of this study was its relatively small and selected group of participants. They will likely be early adopters [79]. Thus we may have overlooked some issues affecting more disadvantaged patients or uninterested health care professionals. Second, EBCD relies strongly on group dynamics and iteration, which may hinder the replicability of our findings. We mitigated these limitations by ensuring the diversity of participants, including some who had experienced critical situations or supported others during such times. Participants also seemed sensitive to the issue of equity as they frequently pointed it out during the interviews and workshops. Finally, the specificities of the health context in Switzerland might limit the transferability of our findings to other settings. However, the basic clinical process of managing and sharing complex information about medications is universal. Thus we are confident that our value propositions can be useful for other settings.

### Implications for Research and Practice

Future research should examine how the coproduction of medication plans changes the management of clinical information and investigate the implications for professional responsibilities and task division [80,81]. In addition, the potential for unintended consequences needs to be studied [82]. Our study’s value propositions could be used in logic models and midrange theories for the implementation and evaluation of medication systems.

Moreover, our value propositions and functionalities should be tested under a variety of conditions, including with diverse, vulnerable groups of medication users and in high-risk situations. Ongoing studies [34,44,63] and a planned proof-of-concept project in Switzerland [45] will provide additional empirical results.

Policy makers and technology vendors must establish the conditions for leveraging the potential of SMP systems to improve medication reconciliation across health care institutions and organizations [83]. In doing so, decision makers must acknowledge the complexity of medication management and invest in adaptable solutions that can accommodate collaboration between health care professionals and patients. We argue for the development of interoperability frameworks enabling the collaborative management of a digital medication plan, with patients as partners. Community Medication Prescription and Dispense profile of Integrating the Healthcare Enterprise [58] supports this by focusing on clinical decisions and treatment planning as its core; however, most public authorities in the world do not currently endorse it. Switzerland’s concept of interoperability in the context of its EPR system is based on the Community Medication Prescription and Dispense profile and Health Level 7 Fast Health care Interoperability Resources specifications [45,57]. The proof of concept and a pilot are currently being implemented by CARA and first volunteering health care providers and their technology providers.

### Conclusions

Modern SMPs should function as digital platforms with adaptable features that facilitate joint medication management and empower patients to be true partners. They should promote and not hinder patient engagement while embracing the shared responsibilities of patients and health care professionals. This shared responsibility should also encompass public health authorities and technological stakeholders, who each play a critical role in creating the conditions for the efficient and safe use of SMPs in daily practice. Introducing SMPs could strengthen partnerships, enhance patient self-management, and improve interprofessional collaboration. SMPs and their use must be tailored to patients’ different levels of health and digital literacy and their personal preferences. The value propositions identified in this study should provide inspiration and guidance for stakeholders and researchers on how to enhance the coproduction of medication management by health care professionals and patients via digital technologies.

## Appendix

Multimedia Appendix 1 Summary and translation of the interview guides.

## Abbreviations

COREQ: Consolidated Criteria for Reporting Qualitative Research
EBCD: experience-based co-design
EPR: electronic patient record
SMP: shared medication plan

## Data Availability

The datasets generated during and analyzed during this study are available from the corresponding author on reasonable request. French versions of the citations are also available upon request.
