# Supplementary material for: Value Propositions for Digital Shared Medication Plans to Boost Patient–Health Care Professional Partnerships: Co-Design Study
Source: J Particip Med. 2025 Jan 28;17:e50828. doi: 10.2196/50828 (PMC11815291; doi:10.2196/50828)
Supplement: Multimedia Appendix 1 [file jopm_v17i1e50828_app1.docx]

# Summary and Translation of the Interview Guides

We prepare with our multidisciplinary research teams and the group of patients as co-researchers. Each focus group was facilitated with one moderator, one assistant and one patient co-researcher. The patient co-researchers had mostly a role for observing and taking personal notes during the discussion to participate actively in the debriefing within the research team. They were also encouraged to ask follow-up questions to stimulate the discussion and to connect with participants to make them feel more accompanied and engage as we assumed they might feel more rapidly comfortable with people of their ages.

|  | **EBCD phase** | **Type of interview** |
| --- | --- | --- |
| 1 | Capturing experiences | Individual interview |
| 2 | Understanding experiences | 1^st^ Focus group |
| 3 | Improving experiences | 2^nd^ Focus group  Focus group with healthcare professionals |
|  |  | 1^st^ EBCD workshop |
| 4 | Recommendations on improving experiences and follow-up | 2^nd^ EBCD workshop |

## Capturing experiences

**Individual interviews**

Objective: get some insights to prepare vignettes for focus groups in addition of key topics from the literature review.

Focus: to describe their experiences in key medication management situations along the patient pathways.

Attitude: let them tell their stories on how they experienced and dealt with situations.

***How do you usually manage your medication plan or treatment list at home?***

- What does it look like?
- Have you ever taken an over-the-counter treatment for an extended period (at least one month)? How did you manage it—pharmacy + doctor?
- And when you visit your general practitioner… how do you prepare to discuss your medications?

***How do you usually manage your medication plan with your general practitioner?***

- Have you ever adjusted a treatment on your own? Under what circumstances? How do you communicate this to your doctor?

***And with the pharmacy, how does it usually go?***

- How do you handle any changes with the pharmacist?
- Are you able to receive any support?

***Have you ever experienced a period where there were significant changes in your medication (e.g., hospitalization, specialist consultation)? Can you tell me about it?***

• How did it go?

- ***Exploring an episode in secondary care with medication changes***

***How was your experience during the initial discussions about your medication?***

- How did you inform them about your usual medications?
- Did you feel that they were aware of and cared about your usual treatments during your care?
- How was your discharge prepared, particularly regarding your medication plan?

***And then, you were discharged… and you went to the pharmacy? How did you feel upon discharge?***

- Were you ready and clear about the medication plan to follow?

***How did things go when you returned home? And the visit to the pharmacy to get your medications?***

- Were the changes clear to you and the pharmacy?
- Time spent? Medication availability?
- How do you perceive and manage the risk of side effects or drug interactions?

***Back home… You find yourself alone with these boxes… How did you experience this?***

- How did the first uses go?
- Were there things that weighed on you? That you were worried about?

***What strategies did you put in place during this period to facilitate these medication changes?***

- To monitor the effectiveness of the treatment and adherence?
- And the evolution of symptoms, such as potential side effects?
- Feedback… with the pharmacy or the doctor?

## Understanding experiences

**1^st^ Focus group**

Focus: experiences of collaboration in medication plan management

Main question: What does it matter to you in medication plan management in collaboration with healthcare professionals?

Plan:

1. Introduction and round tables (15min)
2. Four vignettes* do discuss the main question (1 hour) on four situations :
   - In general (routine)
   - Visit of the general practitioner
   - Journey back home after discharge, up to the visit at the pharmacy
   - Starting a new treatment at home with side effects
3. Final round (10min): with Post-its write down 3 expected added value for introducing a digital shared medication plan.

*Vignettes in French are in the appendix at the end of this document.

## Improving experiences

**2^nd^ Focus group**

Focus: potential improvements and problems that a shared digital tool might bring to support a collaborative management of the medication plan

Plan:

1. Background information about the Swiss EPR and policy context
2. What are the value contributions you are expecting from co-managing a SMP?
   1. Brief presentation of the mind maps synthesizing the Post-its exercise made by everyone at the end of the 1^st^ focus group
3. I wish my digital tool connected to the SMP help me to….
   1. Exploring together situations and related activities: (1) routine, (2) during visits, (3) during care transitions with changes in the medication plan
4. What are the key conditions to achieve a ‘good quality’ co-management?
   1. Barriers? Facilitators?
5. Final round: what do you expect from the co-design workshops with professionals?

**Focus group with professionals**

Focus: exploring the issues of the main touch points (important interaction that matters to patients with healthcare services/professionals) and how a digital patient tool related to a SMP could serve as a facilitator and lead to healthcare improvements.

Main question: What are the key conditions for successfully managing the SMP jointly within the patient’s healthcare journey?

Preparation: the touch points and the added value (mindmap) were intermediary synthesis from the focus groups series with patients.

Plan:

1. Background information about the study, and the context with Swiss EPR
2. Presentation of a synthesis of the added value expected by patients
3. Work among three small groups on 6 touch points to determine the barriers and facilitating role of digital technologies. The 6 touch points were:
   1. Ensure continuity of information and shared responsibility.
   2. Easily manage logistics and administrative tasks to guarantee access to medications.
   3. Plan treatments thoughtfully through discussions leading to shared decision-making.
   4. Monitor treatment use and its effects in preparation for the next consultation.
   5. (Re-)evaluate the entire treatment plan, considering what matters most to the patient.
   6. Learn to live with the illness and medications while benefiting from the necessary support.
4. Presenting flipcharts to summarize and discuss in plenum
5. Final round: what do you expect from the co-design workshops with patients?

**1^st^ EBCD Workshop**

Focus: refine and deepen the synthesis on how to enable the co-management of the medication plan, including with technology, guided by two main questions:

- How can digital tools support co-management? Functions? Features?
- What are the potential avenue of actions to pursue to make it happen?

Plan:

1. Welcoming words and introduction of the workshop (15min)
2. Ice-breaking game in small groups: “what does co-managing a SMP means to you, In one word ?” => participants write the word on a paper and share. (25min)
3. Presentation by the research team with time for feedback/discussions (25min) :
   - - the project and synthesis of the discussions so far.
     - Highlighting that there are goals for the patient, the professionals and collaboration goals.
     - The digital tool should facilitate the work of both patients and professionals to make these collaborations successful and they are many factors influencing the way to reach it.
4. Coffee break (15min)
5. Work in 3 small groups to deepen the two main questions. The “collaboration goals” are distributed among these smaller groups. (45min)
6. Break (10min)
7. Sharing by each group, discussion and wrap-up (50min)

Find below examples of how we illustrated the need for describing how things work out, and facilitate shared understanding on the logic of coproduction, the enabling role of the tool and the consideration:

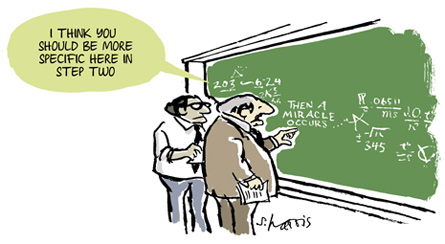


## Recommendations on improving experiences and follow-up

**2nd EBCD workshop**

Focus: validate synthesis, discuss follow-up together and celebrate

Participants received an intermediary report to prepare their feedback or ask their questions.

Plan

1. Welcoming words and presentation of results with room for feedback/questions?
2. Discussions on concrete actions to follow up on the three avenues identified (small group and then plenum)
3. Wrap-up and aperitifs

## Appendix: Vignettes for 1^st^ focus group

**Vignette 1 :**

**En général (routine)**

| Sophie aide sa mère de 88ans qui vit à proximité. Elle prépare son semainier et essaie de suivre la prise de sa dizaine de médicaments. Néanmoins, elle doit de plus en plus négocier… et c’est dur car elle n’arrive pas dire quels sont les traitements les plus importants comme elle ne sait pas vraiment la raison de chacun d’entre eux et «n’est pas du métier». Sophie s’inquiète, elle ira à la prochaine consultation avec sa maman car poser des questions ou demander des informations écrites lui semble difficiles. Un ami à Sophie, qui a une maladie chronique, lui conseille d’écrire un résumé en cas d’urgences ou de nouvelles prises en charge avec, entre autres, les informations sur ses traitements médicamenteux, et, dans la mesure du possible, de tenir à jour un journal avec les changements de traitements et leur justification. « Tout un apprentissage… » |
| --- |

Thèmes identifiés :

- Gestion de l’information par le patient (accessible, actuel, et journal pour l’historique des traitements…).
- Assurer le partage d’information (support,…)
- Compréhension des traitements
- (Apprentissage et confiance)

**Vignette 2 :**

**Lors de la consultation avec le médecin traitant**

| Hélène hésite à changer de médecin traitant. Il est délicat de trouver la bonne combinaison de médicaments et le bon équilibre pour son problème de santé. Les décisions sont difficiles à prendre, et il y a aussi des risques. En plus, elle a l’impression que les 4 spécialistes qu’elle consulte ne travaillent pas comme une équipe, elle doit sans arrêt informer, ré-expliquer, poser les questions, avertir du risque d’interaction ou certaines contre-indication, et d’insister pour, p.ex. éviter des analyses à double, qui sont nécessaires pour ajuster ses traitements. L’autre jour, elle se rend dans une autre pharmacie et doit « se battre » pour obtenir ses médicaments comme indiqué sur l’ordonnance, car même si la combinaison n’est pas habituelle, c’est ce qui fonctionne depuis un bon bout de temps pour elle. Et il y a encore des génériques qui changent…. Bref, parfois, elle a envie de dire « M* » et d’abandonner tous ses efforts… |
| --- |

Thèmes identifiés :

- Efforts et capacité de gestion par le patient => « abandon » qui cause le manque d’adhérence.
- Gestion de l’information pour avoir la même « vue » sur l’historique et les traitements en cours…
- Relation de partenariat avec un engagement souhaité du patient dans la gestion, les décisions, et le suivi.
- Coordination entre les différents intervenants, spécialistes, etc…

**Vignette 3 :**

**Lors d’une hospitalisation avec un retour à domicile (jusqu’au passage à la pharmacie)**

| L’opération délicate de Francis se poursuit avec des complications et 2 mois de convalescences. Déjà en préparation, la marche à suivre liée à ses traitements ne lui semblait pas claire et très standard. Pendant son séjour, il a dû passer dans divers services et institutions. Au moment de rentrer chez lui, il reçoit une lettre fermée à destination de son médecin traitant, et une ordonnance de 2 pages A4 de médicaments sans autres indications. Il est très surpris, il n’avait que 3 médicaments auparavant, il s’empresse donc d’aller chez son médecin traitant. Ensemble ils ont « trié » pour finir avec seulement un traitement supplémentaire.  Quand il partage cette mésaventure avec une amie, elle lui témoigne aussi avoir été confuse en rentrant à domicile, entre ce les traitements qu’elle prenait habituellement et ce qu’on lui a mis sur l’ordonnance. La pharmacie (de garde) n’avait pas pu l’aider, et avait dispensé le tout. |
| --- |

Thèmes identifiés :

- Prise en compte de l’information à l’admission
- Communication patient-institution
- Gestion coordonnée (En cas d’erreur ou discordance identifiée par le patient)
- Compréhension et activation : plan à suivre, signes d’alarmes…
- (Apprentissage et confiance)

**Vignette 4 :**

**À domicile au démarrage d’un nouveau traitement**

| Franck connaît bien sa maladie chronique héréditaire, ses traitements, et il est très actif avec beaucoup de déplacements pour son travail. Il veille à certains symptômes, des risques d’effets indésirables et sur l’efficacité. Il s’est documenté sur les signaux d’alarme. Il vient de changer de traitement anticoagulant, il a rendez-vous dans 3 mois pour faire le point. Il commence à avoir des bleus sur le corps, après s’être informé sur divers sites web et forums, il décide de l’arrêter par lui-même, et d’écrire de suite à son médecin un email qui répond : « vous vous connaissez mieux qu’on vous connaît, on vous fait confiance ». |
| --- |

Thèmes identifiés

- Compréhension et connaissance de sa santé et son corps.
- Connaissance des risques et bénéfices à suivre.
- Décisions autonomes et décisions partagées « à distance ».
